# Spectrum of Microorganisms, Antibiotic Resistance Pattern, and Treatment Outcomes Among Patients With Empyema Thoracis: A Descriptive Cross-Sectional Study From the Bahawal Victoria Hospital Bahawalpur, Punjab, Pakistan

**DOI:** 10.3389/fmed.2021.665963

**Published:** 2021-08-06

**Authors:** Muhammad Atif, Mehwish Naseem, Sajjad Sarwar, Saba Mukhtar, Iram Malik, Muhammad Rauf ul Hassan, Muhammad Nouman Iqbal, Nafees Ahmad

**Affiliations:** ^1^Department of Pharmacy Practice, Faculty of Pharmacy, The Islamia University of Bahawalpur, Bahawalpur, Pakistan; ^2^Department of Pulmonology, Bahawal Victoria Hospital, Bahawalpur, Pakistan; ^3^Rural Health Center, Khanewal, Pakistan; ^4^Department of Pharmacy Practice, Faculty of Pharmacy and Health Sciences, University of Balochistan, Quetta, Pakistan

**Keywords:** drug resistance, antimirobial, antibiotic resisitance, infectious disease, respiratory disease

## Abstract

**Background:** This study involves the analysis of spectrum of microorganisms, antibiotic resistance pattern, and treatment outcomes among empyema thoracis patients. This study also analyzes the factors associated with unsuccessful treatment outcome and duration of hospital stay among the patients.

**Methods:** This was a descriptive, cross-sectional study carried out in the Pulmonology Ward of the Bahawal Victoria hospital, Bahawalpur, Pakistan. All patients with empyema thoracis registered at the study site during the period of 1 year were included in the study. Multivariate regression analysis was used to analyze the factors associated with duration of hospital stay and unsuccessful treatment outcome among the patients.

**Results:** A total 110 patients were included in the study. Most of the patients (*n* = 73, 66.4%) were treated with piperacillin/tazobactam alone and in combination with either one or more than one antibiotics as an empiric therapy. Culture was positive in 58 (52.7%) patients and the most commonly identified organisms included, gram-negative *Pseudomonas aeruginosa* (*n* = 20; 18.8%) and *Klebsiella sp*. (*n* = 11, 10%) followed by same proportion of *E. coli*. The most commonly identified bacterial isolates showed high level of resistance against antibiotics used as an empiric therapy, while these showed low level of resistance against amoxicillin, clarithromycin, ertapenem, colistin, tigecycline, fosfomycin, rifampicin, and vancomycin. In this study, 82 (74.5%) patients successfully completed the treatment, while 12 (11%) showed no clinical improvement, 5 (4.5%) lost to follow up and 11 (10%) died. In multivariate binary logistic regression analysis, none of the patient attributes were significantly associated with unsuccessful treatment outcome, while in multivariate linear regression analysis, the factors which were significantly associated with duration of hospital stay included; duration of symptoms <2 weeks prior to admission (*p* = 0.008, beta = −0.247) and resistance to five antibiotic classes (*p* = 0.02, beta = 0.280).

**Conclusion:** Close to 25% of the patients did not complete the treatment successfully. Most of the common bacterial isolates showed high level of resistance against the broad-spectrum antibiotics used as an empiric therapy. This is alarming. However, better sensitivity of common bacterial isolates against standardized first line treatment for empyema thoracis is promising.

## Introduction

Empyema thoracis is an infectious disease which causes the accumulation of frank pus in the pleural space of lungs (1). It mostly appears as a complication of hospital and community acquired pneumonia, however, it also occurs due to other causes like thoracic injuries, chest trauma, bronchogenic carcinoma, esophageal rupture, immune-compromised states, and other postsurgical infections (1, 2). The clinical sign and symptoms of empyema include; pleuritic chest pain, cough, fever, chills, weight loss, anorexia, dyspnea, and night sweats (1, 3). The diagnosis of empyema is established by the presence of pus and fluid in the pleural space followed by microbiological assay of pleural fluid, while gene expert and acid fast bacilli (AFB) smear examination are used for the detection of *Mycobacterium tuberculosis* (2). The major aim of empyema treatment is to eliminate the infection and re-expansion of the lungs which is usually achieved by eradicating the bacterial growth from the pleural fluid by the use of appropriate antibiotic therapy along with drainage process (1, 2, 4, 5).

Epidemiological data about empyema thoracis is limited. However, over the last few years, the incidence rate of this disease is increasing globally, whereas, developing countries accounts for high burden of empyema thoracis (2, 6–8). Each year, in the United States (US), ~1 million patients were hospitalized with pneumonia. Among those hospitalized patients, 20–40% had para pneumonic effusion, while 5–10% of these patients progressed to empyema thoracis (2). Unfortunately, very scarce data on empyema thoracis is available from Pakistan. According to a study conducted in Pakistan, the empyema thoracis is a complication that accounts 18% of thoracic injuries (9).

The leading pathogens in community acquired empyema include, gram-negative *Escherichia coli (E.coli), Klebsiella pneumoniae*, and gram-positive *Staphylococcus* and *Streptococcus species*, while, in hospital acquired empyema, the most common pathogens are gram-negative *Pseudomonas, Enterobacter species*, and *Methicillin resistant Staphylococcus aureus (MRSA)* (1). All these pathogens causing empyema are the same well-known pathogens causing community and hospital acquired infection, worldwide (10–15). Moreover, *Mycobacterium tuberculosis* is also a common pathogen which may cause empyema (16). In case, microbiology reports are not available, the differential diagnosis can be made on patient history (17).

According to the American Association for Thoracic Surgery (AATS) consensus guidelines for the management of empyema, the diseases should be treated initially by drainage process along with antibiotics (2). The guidelines further suggest that for community acquired empyema thoracis, cephalosporins like ceftriaxone along with metronidazole, or amino-penicillin with beta lactamase inhibitors (e.g., ampicillin/sulbactam) should be preferred. While, vancomycin along with Cefepime and metronidazole or with piperacillin/tazobactam should be used for hospital acquired empyema (2). Despite these defined treatment recommendations, achievement of treatment success and reduction in mortality rates among empyema thoracis patients continues to be a problem for healthcare professionals, predominantly due to antibiotic resistance (18, 19). Unfortunately, antibiotic resistance is much higher in developing countries, for example, in India and Pakistan, due to common inappropriate use of antibiotics (20–22). Importantly, a change in the pathogen profile and pattern of susceptibility has been observed in different countries against antimicrobials, and it changes over time (23, 24). This phenomena necessitates that antibiotic resistance and sensitivity pattern should be observed over time for infectious diseases (24).

Despite of having a knowledge about increasing incidence of this disease, only few studies (25, 26) were conducted in Pakistan among empyema thoracis patients. However, the pattern of antibiotic resistance and final treatment outcomes were not addressed in depth. Moreover, patient's attributes associated with final treatment outcome and length of hospital stay are still under investigation in Pakistan. Therefore, the aim of this study was to investigate the clinical characteristics, spectrum of microorganisms, antibiotic resistance pattern, and treatment outcomes among empyema thoracis patients in Pakistan. Moreover, we also analyzed the independent factors associated with unsuccessful treatment outcome and duration of hospital stay among the patients.

## Materials and Methods

### Study Setting

This study was conducted in the chest disease unit (CDU) of the Bahawal Victoria hospital (BVH), Bahawalpur, Punjab, Pakistan. This tertiary care hospital has a capacity of over 1,600 beds (27). The CDU serves both indoor and outdoor patients suffering from different forms of lung diseases. The CDU has five to six consultants, 15–18 doctors, two pharmacists and has a bed capacity of 64 (27).

### Study Design and Study Population

**Figure 1:**
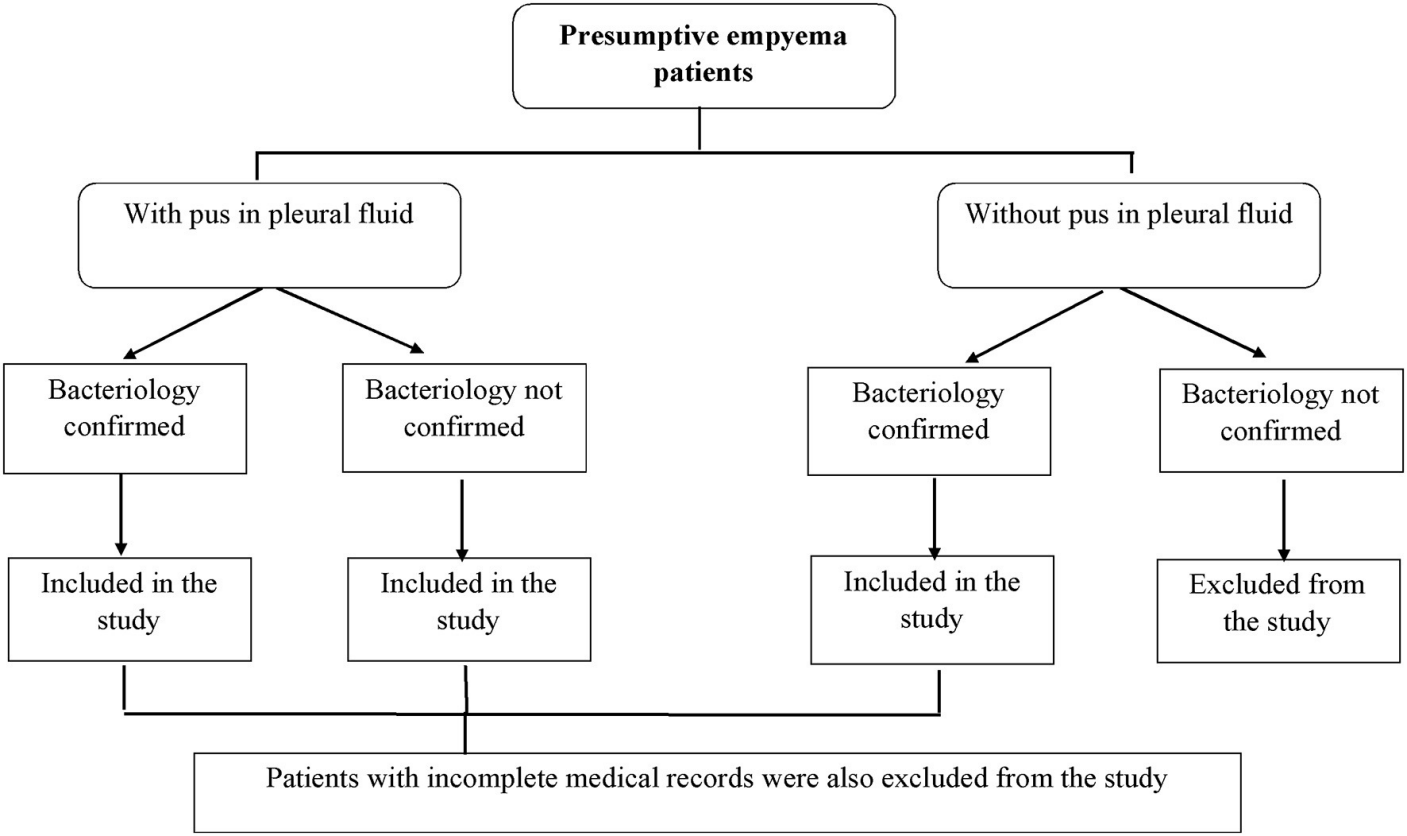
Description of the patients who met the inclusion criteria.

This was a descriptive cross-sectional study. A detailed information about the patients who met the inclusion criteria is presented in Figure 1.

### Microbiological Examination

The BVH follows standard operating procedures for microbiological examination of culture specimens (2). For empyema patients, the specimen (pleural fluid) for culture was taken via drainage technique (thoracentesis). The specimen was drawn from freshly drained fluid to avoid the contamination and then inoculated in the aerobic and anaerobic sterile standard culture bottles (BACTEC culture bottles) for culture and staining (2, 28). The specimen was then incubated in the BACTEC960 culture bottles at 35°C for 2–3 days. Gram staining, gene expert and AFB smear examination (for *M. Tuberculosis*) were performed on the culture positive bottles and the culture was examined daily to check the growth of microorganisms. The identification of organism and DST was performed by subculture technique in a suitable solid media (24, 28). Standard methodology for characterization of pathogens and antibiotic concentrations in DST were used.

### Diagnosis and Treatment of Patients

In this study setting, presumptive patients of empyema were diagnosed through clinical presentation of patients (i.e., pleuritic chest pain, cough, fever, chills, weight loss, anorexia, dyspnea, and night sweats), pleural ultrasound (US), conventional chest x-ray (CXR), computed tomography (CT-scan) and pleural fluid analysis (1, 2). Immediately after clinical diagnosis patients were started on empiric therapy. Once pleural fluid analysis (i.e., microbiological analysis) reports became available, the empiric therapy was replaced with appropriate modified regimen. In case, patient's culture was negative (only for those who had pus in pleural fluid), the patient was still considered as clinically diagnosed case and asked to complete the empyema treatment (29). Pleural fluid drainage was done on the basis of clinician's suggestion for the improvement of patient's condition (2).

### Outcomes Variables

Outcome variables included; spectrum of microorganisms, antibiotic resistance pattern and treatment outcomes.

Treatment outcomes were broadly divided into following categories; treatment completed with clinical improvement, no clinical improvement, loss to follow up and death.

#### Treatment Completed With Clinical Improvement

The patients with empyema thoracis who completed treatment duration of 2–6 weeks with clinical improvement. Such patients were classified as successfully treated patients (2, 6).

#### No Clinical Improvement

The patients with empyema thoracis who did not improve at any stage of treatment.

#### Loss to Follow-Up

The patients with empyema thoracis who left the hospital on their own before treatment completion (27).

#### Death

Patients died during the treatment.

#### Unsuccessful Outcome

No clinical improvement, loss to follow up due to any reason and death during treatment were broadly classified as unsuccessful treatment outcome.

### Data Collection

The study cohort included all those confirmed cases of empyema thoracis who were registered at the study site from October, 2019 to September, 2020. The data collection form was designed through literature review (5, 6, 30). It consisted of information on respondent's sociodemographic and clinical characteristics, antibiotic sensitivity and resistance pattern, treatment regimen, duration of symptoms, duration of hospital stay, comorbidities, and final treatment outcomes. Microbiological reports and medical records of the patients were used to obtain patient data.

### Data Analysis

Data analysis was performed by using Statistical Package for Social Sciences (IBM SPSS statistics for windows version 20.0, Armonk, NY: IBM Corp) (27). Continuous variables were presented as mean and standard deviation (SD), whereas counts (n) and proportions (%) were used to present categorical variables (27). Multivariate binary logistic regression analysis was used to identify the factors associated with unsuccessful treatment outcomes and multivariate linear regression analysis was used to identify the factors associated with duration of hospital stay. The variable with a *p* < 0.05 in univariate analysis were entered into multivariate analysis (27).

### Ethical Approval

Pharmacy research and ethics committee (PREC) at the Islamia university of Bahawalpur (Reference no: 109-2020-/PREC) approved the design and conduct of the study.

## Results

### Description of the Patients

At the time of data collection, 140 presumptive cases of empyema thoracis were reported at the study site, while, 110 cases of empyema thoracis were finely included in the study. Thirty patients with no pus in the pleural fluid (with no bacterial growth) (*n* = 20) and the patients with incomplete medical records (*n* = 10) were excluded from the study.

### Sociodemographic and Clinical Characteristics of the Patients

Out of the total 110 patients, 75 (68.2%) were male, 70 (63.6%) were resident of rural areas, and 50 (45.4%) were illiterate. The mean age of the patients was 39.3 (SD = 16.6) years. Out of the total, 71 (64.5%) patients were not exposed to biomass fuel. The community acquired empyema was diagnosed in 82 (74.5%) patients. A total of 58 (52.7%) patients were culture positive, whereas, 48 (43.6%) were culture negative. Close to half of the patients (46.4%) had symptom duration of 2–4 weeks. The mean duration of hospital stay was 2.3 (SD = 1.4) weeks (Table 1).

**Table 1:** Sociodemographic and clinical characteristics of the patients (*N* = 110).

**Characteristics**	**Patients, *n* (%)**	**Characteristics**	**Patients, *n* (%)**
**Age (years), mean** **±** **SD (39.3** **±** **16.6)**		**Drug sensitivity test**	
5–14	3 (3)	Yes	106 (96.4)
15–24	20 (18.1)	No	4 (3.6)
25–35	26 (24)	**Microorganism growth**	
35–44	12 (11)	Positive	58 (53)
45–54	25 (23)	Negative	48 (43.6)
55–64	16 (14.5)	Not tested	4 (3.6)
>65	8 (7.2)	**Microorganism involved**	
**Gender**		Monomicrobial	50 (45.5)
Male	75 (68.2)	Polymicrobial	8 (7.3)
Female	35 (31.8)	Not tested	4 (3.6)
**Residence**		No growth seen	48 (43.6)
Urban	40 (36.4)	**Improvement in symptoms**	
Rural	70 (63.6)	Improved after modification	40 (36.4)
**Smoking**		Improved on empiric therapy	47 (42.7)
Current smoker	9 (8.1)	Not improved	23 (20.9)
Ex-smoker	18 (16.4)	**Duration of symptoms**	
Non-smoker	83 (75.5)	<2 weeks	28 (25.5)
**Biomass fuel exposure**		2–4 weeks	51 (46.4)
Yes	39 (35.5)	More than 4 weeks	31 (28.2)
No	71 (64.5)	**Duration of hospital stay (weeks), mean** **±** **SD (2.3** **±** **1.4)**	
**Education level**		< weeks	30 (27.3)
Illiterate	50 (45.4)	2–4 weeks	76 (69.1)
Primary school	20 (18.1)	More than 4 weeks	4 (3.6)
Secondary school	27 (24.5)	**Comorbidities**	
Higher secondary school/diploma	9 (8.1)	Hypertension	4 (3.6)
University graduates	3 (2.7)	Diabetes mellitus	25 (22.7)
Others	1 (0.9)	Cardiovascular disease	1 (0.9)
**Work status**		Anemia	1 (0.9)
Employed	2 (1.8)	Hepatitis	3 (2.7)
Self-employed	60 (54.5)	Asthma	3 (2.7)
Unemployed	15 (13.6)	COPD	4 (3.6)
Student	9 (8.2)	Bronchiectasis	4 (3.6)
Retired	3 (2.7)	Cancer	3 (2.7)
Others	21 (19.1)	Arthritis	2 (1.8)
**Diagnosis**		Goiter	1 (0.9)
Hospital acquired empyema	23 (21)	None	65 (59.1)
Community acquired empyema	82 (74.5)		
Tuberculous empyema	5 (4.5)		

*COPD, chronic obstructive pulmonary disease*.

### Empiric Therapy Started Among the Patients

At the beginning of therapy, 73 (66.4%) patients were treated with piperacillin+ tazobactam; alone in 14 (12.7%) cases and in combination with moxifloxacin 26 (23.6%) followed by linezolid 9 (8.1%) and ceftriaxone 6 (5.4%). Remaining 37 (34%) patients were treated with other antibiotics used alone or in combination which included; moxifloxacin (*n* = 56; 50.9%), ceftriaxone (*n* = 37; 33.6%), linezolid (*n* = 17; 15.5%), vancomycin (*n* = 10; 9.1%), ciprofloxacin (*n* = 8; 7.3%), clarithromycin (*n* = 5; 4.5%), imipenem (*n* = 5; 4.5%) and levofloxacin (*n* = 1; 0.9%).

### Organisms Isolated From the Specimen

**Figure 2:**
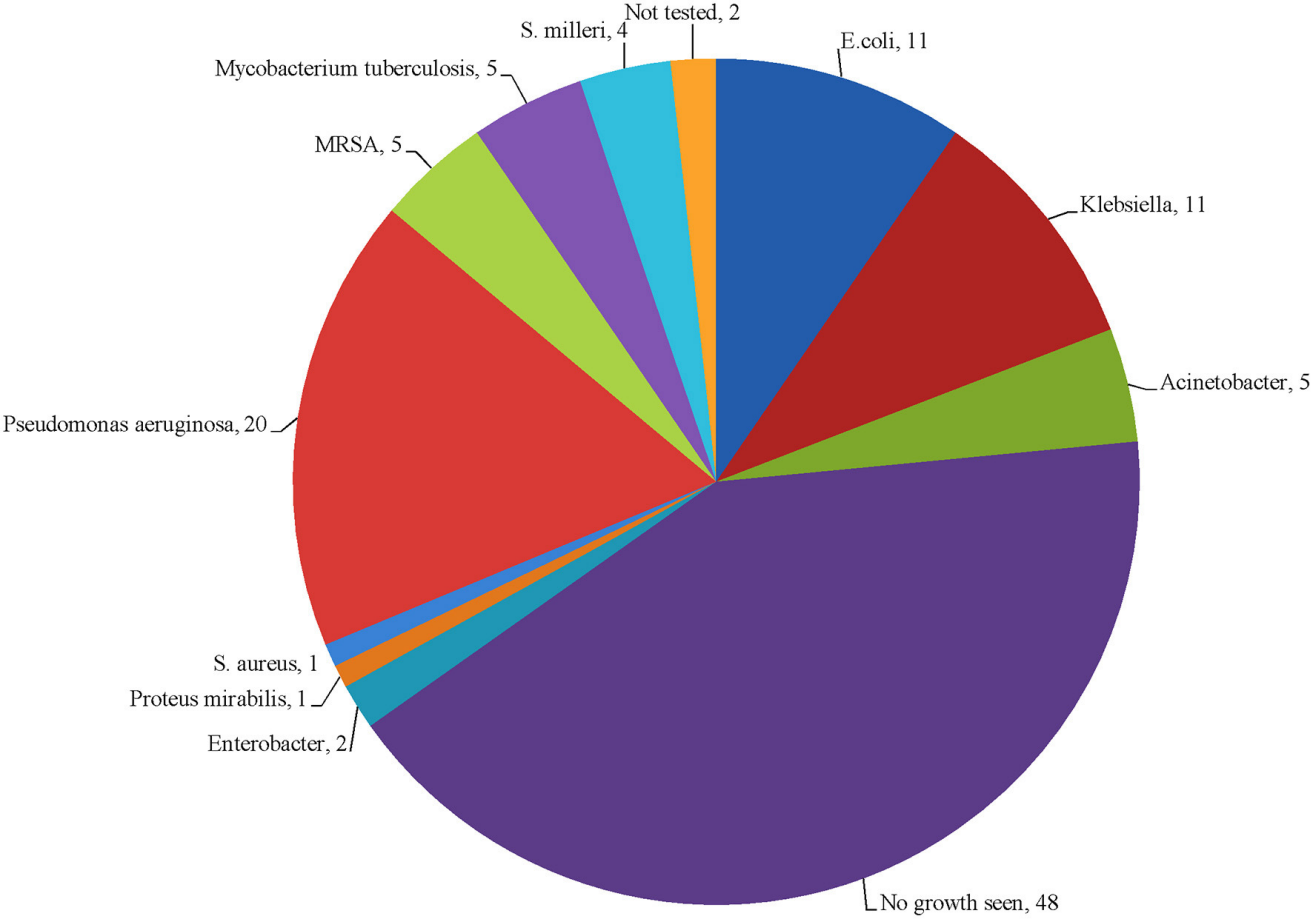
Number and type of bacterial species identified in the study.

Culture yielded a total of 65 bacterial isolates from 58 (52.7%) culture positive specimens. Out of these 58 specimens, 50 (86.2%) were mono-microbial and 8 (13.7%) were poly-microbial. In 48 (43.6%) specimens, there was no growth of microorganism, while 4 (3.6%) specimens were not tested for culture growth. The isolated organisms were categorized as gram-negative (*n* = 50, 43.4%), gram-positive (*n* = 10, 9%), and *Mycobacterium tuberculosis* (*n* = 5, 4.3%). Among them, *Pseudomonas aeruginosa* was dominant 20 (18.8%), while *E. coli and Klebsiella sp*. shared same proportions (*n* = 11, 10%). The type of bacterial species identified in this study is presented in Figure 2.

### Antibiotic Sensitivity and Resistance Pattern Against Bacterial Isolates

Out of the total 65 isolates, piperacillin/tazobactam was tested in 40 isolates (gram-negative = 32, gram-positive = 8). Out of these, nine (22.5%) gram-negative and one (2.5%) gram-positive bacterial isolates were resistant to aforementioned antibiotic. Moxifloxacin was tested in 37 isolates (gram-negative = 29, gram-positive = 8). Out of these, six (16.2%) gram-negative isolates were resistant to moxifloxacin. Similarly, ceftriaxone was tested in 38 isolates (gram-negative = 30, gram-positive = 8), from which 13 (34.2%) gram-negative isolates were resistant to above mentioned antibiotic. Amoxicillin/clavulanic acid, clarithromycin, colistin, vancomycin, ertapenem, and tigecycline showed better sensitivity against bacterial isolates (Table 2). A detailed information about the number of drugs resistant to each bacterial isolate is mentioned in Supplementary Table 1.

**Table 2:** Antibiotic sensitivity and resistance against organism isolates (*N* = 110).

**Antibiotic tested**	**Gram negative**		**Gram positive**	
	***R* (%)**	***S* (%)**	***R* (%)**	***S* (%)**
Amoxicillin + clavulanic acid (*n =* 38)	12 (31.5)	18 (47.3)	0 (0)	8 (21.0)
Piperacillin + tazobactam (*n =* 40)	9 (22.5)	23 (57.5)	1 (2.5)	7 (17.5)
Oxacillin (*n =* 35)	1 (2.8)	26 (74.2)	4 (11.4)	4 (11.4)
Ampicillin (*n =* 34)	2 (5.8)	25 (73.5)	0 (0)	7 (20.5)
Penicillin (*n =* 36)	3 (8.3)	26 (72.2)	0 (0)	7 (19.4)
Amoxicillin (*n =* 34)	1 (2.9)	25 (73.5)	0 (0)	8 (23.5)
Aztreonam (*n =* 35)	5 (14.2)	22 (62.8)	0 (0)	8 (22.8)
Imipenem (*n =* 40)	10 (25)	22 (55)	0 (0)	8 (20)
Meropenem (*n =* 42)	11 (26.1)	23 (54.7)	0 (0)	8 (19.0)
Ertapenem (*n =* 33)	1 (3.0)	25 (75.7)	0 (0)	7 (20)
Cefuroxime (*n =* 33)	5 (15.1)	21 (63.6)	0 (0)	7 (21.2)
Cefaclor (*n =* 35)	3 (8.5)	24 (68.5)	0 (0)	8 (22.8)
Cefixime (*n =* 38)	11 (28.9)	19 (50)	0 (0)	8 (21.0)
Cefepime (*n =* 40)	11 (27.5)	21 (52.5)	0 (0)	8 (20)
Ceftazidime (*n =* 40)	13 (32.5)	19 (47.5)	0 (0)	8 (20)
Cefotaxime (*n =* 41)	15 (36.5)	18 (43.9)	0 (0)	8 (19.5)
Cefpodoxime (*n =* 36)	2 (5.5)	26 (72.2)	0 (0)	8 (22.2)
Ceftriaxone (*n =* 38)	13 (34.2)	17 (44.7)	0 (0)	8 (21.0)
Cephalexin (*n =* 35)	2 (5.7)	25 (71.4)	0 (0)	8 (22.8)
Cephradine (*n =* 36)	4 (11.1)	24 (66.6)	0 (0)	8 (22.2)
Ciprofloxacin (*n =* 44)	22 (50)	14 (31.8)	3 (6.8)	5 (11.3)
Moxifloxacin (*n =* 37)	6 (16.2)	23 (62.1)	0 (0)	8 (21.6)
Levofloxacin (*n =* 42)	7 (16.6)	27 (64.2)	0 (0)	8 (19.0)
Ofloxacin (*n =* 37)	7 (18.9)	22 (59.4)	0 (0)	8 (21.6)
Enoxacin (*n =* 34)	1 (2.9)	25 (73.5)	0 (0)	8 (23.5)
Sparfloxacin (*n =* 33)	1 (3.0)	25 (75.7)	0 (0)	7 (21.2)
Nalidixic acid (*n =* 34)	2 (5.8)	24 (70.5)	0 (0)	8 (23.5)
Amikacin (*n =* 43)	11 (25.5)	24 (55.8)	0 (0)	8 (18.6)
Gentamycin (*n =* 37)	16 (43.2)	14 (37.8)	0 (0)	7 (18.9)
Tobramycin (*n =* 38)	5 (13.1)	25 (65.7)	0 (0)	8 (21.0)
Sulphamethoxazole (*n =* 32)	6 (18.7)	19 (59.3)	2 (6.25)	5 (15.6)
Chloramphenicol (*n =* 34)	1 (2.9)	25 (73.5)	0 (0)	8 (23.5)
Tetracycline (*n =* 35)	2 (5.7)	25 (71.4)	0 (0)	8 (22.8)
Doxycycline (*n =* 40)	5 (12.5)	28 (70)	0 (0)	7 (17.5)
Colistin (*n =* 35)	0 (0)	27 (77.1)	0 (0)	8 (22.8)
Septran (*n =* 35)	2 (5.7)	25 (71.4)	0 (0)	8 (22.8)
Clindamycin (*n =* 35)	3 (8.5)	24 (68.5)	1 (2.8)	7 (20)
Clarithromycin (*n =* 34)	1 (2.9)	25 (73.5)	0 (0)	8 (23.5)
Vancomycin (*n =* 36)	1 (2.7)	27 (75)	0 (0)	8 (13.8)
Erythromycin (*n =* 35)	3 (8.5)	24 (68.5)	4 (11.4)	4 (11.4)
Linezolid (*n =* 39)	5 (12.8)	26 (66.6)	0 (0)	8 (20.5)
Fusidic acid (*n =* 35)	1 (2.8)	26 (74.2)	1 (2.8)	7 (20)
Fosfomycin (*n =* 34)	1 (2.9)	25 (73.5)	0 (0)	8 (23.5)
Tigecycline (*n =* 34)	1 (2.9)	25 (73.5)	0 (0)	8 (23.5)
Cefoperazone+ sulbactam (*n =* 40)	7 (17.5)	25 (73.5)	0 (0)	8 (23.5)
Pipemidic acid (*n =* 34)	1 (2.9)	25 (73.5)	0 (0)	8 (23.5)
Norfloxacin (*n =* 34)	1 (2.9)	25 (73.5)	0 (0)	8 (23.5)
Rifampicin (*n =* 34)	1 (2.9)	25 (73.5)	0 (0)	8 (23.5)
Cotrimoxazole (*n =* 34)	3 (8.8)	23 (67.6)	0 (0)	8 (23.5)

*Calculations were based on number of specimens with positive organism growth in which specific antibiotics were tested for sensitivity and resistance, R, resistance; S, sensitivity*.

### Percentage Resistance of Organism Isolates Against Antibiotic

Among most common gram-negative bacterial isolates (i.e., *E. coli, Klebsiella sp., and Acinetobacter species*) identified in this study, highest level of resistance was seen against third generation cephalosporins (i.e., ceftriaxone, cefotaxime), piperacillin/tazobactam, imipenem, and second, third and fourth generation fluoroquinolones. However, amoxicillin/clavulanic acid, ertapenem, clarithromycin, colistin, vancomycin, fosfomycin, and tigecycline showed low level of resistance against most common gram-negative isolates (Table 3). Drug resistance index of *E. coli was 0.094 and 0.86 during first and second 6 months, respectively. In case of Klebsiella sp*., drug resistance index of 0.16 and 0.7 was observed *during first and second 6 months, respectively*. Drug resistance index *of Pseudomonas aeruginosa was 0.065 and 0.23 during first and second 6 months, respectively. Details about* drug resistance index of *E. coli, Klebsiella sp*., and *Pseudomonas aeruginosa* are provided in Supplementary Table 2.

**Table 3:** Percentage resistance of organism isolates against antibiotics (*N* = 110).

**Antibiotics**	***E-coli***		***Klebsiella specie***		***Acinetobacter***		***P. aeruginosa***		***MRSA***		***Mycobacterium TB***		***S. milleri***		***Enterobacter***		***Proteus mirabilis***	
	**R/(R+S)**	**R%**	**R/(R+S)**	**R%**	**R/(R+S)**	**R%**	**R/(R+S)**	**R%**	**R/(R+S)**	**R%**	**R/(R+S)**	**R%**	**R/(R+S)**	**R%**	**R/(R+S)**	**R%**	**R/(R+S)**	**R%**
Amoxicillin+ clavulanic acid	5/(5+3)	62.5	4/(4+3)	57.1	1/(1+2)	33.3	1/(1+9)	10	0/(0+4)	0	0/(0+1)	0	0/(0+4)	0	1/(1+0)	100	0/(0+1)	0
Piperacillin+ tazobactam	2/(2+5)	28.5	2/(2+3)	40	3/(3+1)	75	2/(2+12)	14.2	1/(1+3)	25	0/(0+1)	0	0/(0+4)	0	0/(0+1)	0	0/(0+1)	0
Oxacillin	0/(0+7)	0	1/(1+4)	20	0/(0+2)	0	0/(0+11)	0	4/(4+0)	100	0/(0+1)	0	0/(0+4)	0	0/(0+1)	0	0/(0+1)	0
Ampicillin	0/(0+7)	0	1/(1+4)	20	0/(0+2)	0	0/(0+10)	0	0/(0+4)	0	0/(0+1)	0	0/(0+3)	0	1/(1+1)	50	0/(0+1)	0
Penicillin	0/(0+7)	0	2/(2+4)	33.3	1/(1+2)	33.3	0/(0+11)	0	0/(0+4)	0	0/(0+1)	0	0/(0+3)	0	0/(0+1)	0	0/(0+1)	0
Amoxicillin	0/(0+7)	0	1/(1+4)	20	0/(0+2)	0	0/(0+10)	0	0/(0+4)	0	0/(0+1)	0	0/(0+4)	0	0/(0+1)	0	0/(0+1)	0
Aztreonam	2/(2+6)	25	2/(2+3)	40	0/(0+2)	0	0/(0+10)	0	0/(0+4)	0	0/(0+1)	0	0/(0+4)	0	1/(1+0)	100	0/(0+1)	0
Imipenem	2/(2+5)	28.5	3/(3+2)	60	3/(3+1)	75	2/(2+12)	14.2	0/(0+4)	0	0/(0+1)	0	0/(0+4)	0	0/(0+1)	0	0/(0+1)	0
Meropenem	2/(2+7)	22.2	3/(3+4)	42.8	3/(3+0)	100	3/(3+10)	23.0	0/(0+4)	0	0/(0+1)	0	0/(0+4)	0	0/(0+1)	0	0/(0+1)	0
Ertapenem	0/(0+7)	0	1/(1+4)	20	0/(0+2)	0	0/(0+10)	0	0/(0+4)	0	0/(0+1)	0	0/(0+3)	0	0/(0+1)	0	0/(0+1)	0
Cefuroxime	2/(2+5)	28.5	2/(2+3)	40	0/(0+2)	0	0/(0+10)	0	0/(0+4)	0	0/(0+1)	0	0/(0+3)	0	1/(1+0)	100	0/(0+1)	0
Cefixime	2/(2+6)	25	3/(3+3)	50	2/(2+1)	66.6	3/(3+8)	27.2	0/(0+4)	0	0/(0+1)	0	0/(0+4)	0	1/(1+0)	100	0/(0+1)	0
Cefaclor	1/(1+6)	14.2	1/(1+4)	20	1/(1+2)	33.3	0/(0+10)	0	0/(0+4)	0	0/(0+1)	0	0/(0+4)	0	0/(0+1)	0	0/(0+1)	0
Cefepime	1/(1+6)	14.2	1/(1+4)	20	2/(2+2)	50	7/(7+7)	50	0/(0+0)	0	0/(0+0)	0	0/(0+4)	0	0/(0+1)	0	0/(0+1)	0
Ceftazidime	1/(1+6)	14.2	1/(1+4)	20	4/(4+0)	100	7/(7+7)	50	0/(0+4)	0	0/(0+1)	0	0/(0+4)	0	0/(0+1)	0	0/(0+1)	0
Cefotaxime	5/(5+4)	55.5	5/(5+2)	71.4	3/(3+1)	75	1/(1+10)	9.0	0/(0+4)	0	0/(0+1)	0	0/(0+4)	0	0/(0+1)	0	1/(1+0)	100
Cefpodoxime	1/(1+6)	14.2	1/(1+6)	14.2	0/(0+2)	0	0/(0+10)	0	0/(0+4)	0	0/(0+1)	0	0/(0+4)	0	0/(0+1)	0	0/(0+1)	0
Ceftriaxone	4/(4+4)	50	3/(3+4)	42.8	2/(2+1)	66.6	2/(2+8)	20	0/(0+4)	0	0/(0+1)	0	0/(0+4)	0	1/(1+0)	100	1/(1+0)	100
Cephalexin	0/(0+7)	0	1/(1+4)	20	1/(1+2)	33.3	0/(0+10)	0	0/(0+4)	0	0/(0+1)	0	0/(0+4)	0	0/(0+1)	0	0/(0+1)	0
Cephradine	2/(2+6)	25	2/(2+4)	33.3	0/(0+2)	0	0/(0+10)	0	0/(0+4)	0	0/(0+1)	0	0/(0+4)	0	0/(0+1)	0	0/(0+1)	0
Ciprofloxacin	5/(5+3)	62.5	6/(6+1)	85.7	3/(3+1)	75	6/(6+8)	42.8	3/(3+1)	75	0/(0+1)	0	0/(0+4)	0	1/(1+1)	50	1/(1+0)	100
Moxifloxacin	2/(2+6)	25	1/(1+4)	20	2/(2+2)	50	1/(1+9)	10	0/(0+4)	0	0/(0+1)	0	0/(0+4)	0	0/(0+1)	0	0/(0+1)	0
Levofloxacin	1/(1+6)	14.2	1/(1+6)	14.2	2/(2+2)	50	3/(3+11)	21.4	0/(0+4)	0	0/(0+1)	0	0/(0+4)	0	0/(0+1)	0	0/(0+1)	0
Ofloxacin	2/(2+5)	28.5	1/(1+4)	20	2/(2+1)	66.6	2/(2+10)	16.6	0/(0+4)	0	0/(0+1)	0	0/(0+4)	0	0/(0+1)	0	0/(0+1)	0
Enoxacin	0/(0+7)	0	1/(1+4)	20	0/(0+2)	0	0/(0+10)	0	0/(0+4)	0	0/(0+1)	0	0/(0+4)	0	0/(0+1)	0	0/(0+1)	0
Sparfloxacin	0/(0+7)	0	1/(1+4)	20	0/(0+2)	0	0/(0+10)	0	0/(0+4)	0	0/(0+1)	0	0/(0+3)	0	0/(0+1)	0	0/(0+1)	0
Nalidixic acid	0/(0+7)	0	2/(2+3)	40	0/(0+2)	0	0/(0+10)	0	0/(0+4)	0	0/(0+1)	0	0/(0+4)	0	0/(0+1)	0	0/(0+1)	0
Amikacin	0/(0+8)	0	4/(4+3)	57.1	2/(2+2)	50	5/(5+9)	35.7	0/(0+4)	0	0/(0+1)	0	0/(0+4)	0	0/(0+1)	0	0/(0+1)	0
Gentamycin	2/(2+5)	28.5	3/(3+2)	60	3/(3+0)	100	6/(6+7)	46.1	1/(1+3)	25	0/(0+1)	0	0/(0+4)	0	1/(1+0)	100	1/(1+0)	100
Tobramycin	1/(1+6)	14.2	1/(1+4)	20	0/(0+3)	0	3/(3+10)	23.0	0/(0+4)	0	0/(0+1)	0	0/(0+4)	0	0/(0+1)	0	0/(0+1)	0
Sulphamethoxazole	2/(2+5)	28.5	1/(1+4)	20	2/(2+0)	100	1/(1+8)	11.1	2/(2+2)	50	0/(0+1)	0	0/(0+3)	0	0/(0+1)	0	0/(0+1)	0
Chloramphenicol	0/(0+7)	0	1/(1+4)	20	0/(0+2)	0	0/(0+10)	0	0/(0+4)	0	0/(0+1)	0	0/(0+4)	0	0/(0+1)	0	0/(0+1)	0
Tetracycline	0/(0+7)	0	1/(1+4)	20	0/(0+2)	0	1/(1+10)	9.0	0/(0+4)	0	0/(0+1)	0	0/(0+4)	0	0/(0+1)	0	0/(0+1)	0
Doxycycline	1/(1+7)	12.5	1/(1+6)	14.2	1/(1+3)	25	1/(1+10)	9.0	0/(0+4)	0	0/(0+1)	0	0/(0+3)	0	1/(1+1)	50	0/(0+1)	0
Colistin	0/(0+7)	0	0/(0+4)	0	0/(0+2)	0	0/(0+12)	0	0/(0+4)	0	0/(0+1)	0	0/(0+4)	0	0/(0+1)	0	0/(0+1)	0
Trimethoprim and sulfamethoxazole	1/(1+7)	12.5	1/(1+4)	20	0/(0+2)	0	0/(0+10)	0	0/(0+4)	0	0/(0+1)	0	0/(0+4)	0	0/(0+1)	0	0/(0+1)	0
Clindamycin	1/(1+6)	14.2	2/(2+3)	40	0/(0+2)	0	0/(0+11)	0	0/(0+4)	0	0/(0+1)	0	1/(1+3)	25	0/(0+1)	0	0/(0+1)	0
Clarithromycin	0/(0+7)	0	1/(1+4)	20	0/(0+2)	0	0/(0+10)	0	0/(0+4)	0	0/(0+1)	0	0/(0+4)	0	0/(0+1)	0	0/(0+1)	0
Vancomycin	0/(0+7)	0	1/(1+4)	20	0/(0+2)	0	0/(0+11)	0	0/(0+4)	0	0/(0+1)	0	0/(0+4)	0	0/(0+2)	0	0/(0+1)	0
Erythromycin	1/(1+6)	14.2)	1/(1+4)	20	0/(0+2)	0	1/(1+10)	9.0	3/(3+1)	75	0/(0+1)	0	1/(1+3)	25	0/(0+1)	0	0/(0+1)	0
Linezolid	2/(2+6)	25	3/(3+4)	42.8	0/(0+2)	0	0/(0+11)	0	0/(0+4)	0	0/(0+1)	0	0/(0+4)	0	0/(0+2)	0	0/(0+1)	0
Fusidic acid	0/(0+7)	0	1/(1+4)	20	0/(0+2)	0	0/(0+11)	0	1/(1+3)	25	0/(0+1)	0	0/(0+4)	0	0/(0+1)	0	0/(0+1)	0
Fosfomycin	0/(0+7)	0	1/(1+4)	20	0/(0+2)	0	0/(0+10)	0	0/(0+4)	0	0/(0+1)	0	0/(0+4)	0	0/(0+1)	0	0/(0+1)	0
Tigecycline	0/(0+7)	0	1/(1+4)	20	0/(0+2)	0	0/(0+10)	0	0/(0+4)	0	0/(0+1)	0	0/(0+4)	0	0/(0+1)	0	0/(0+1)	0
Cefoperazone+ sulbactam	1/(1+8)	11.1	3/(3+4)	42.8	2/(2+1)	66.6	1/(1+10)	9.0	0/(0+4)	0	0/(0+1)	0	0/(0+4)	0	0/(0+1)	0	0/(0+1)	0
Pipemidic acid	0/(0+7)	0	1/(1+4)	20	0/(0+2)	0	0/(0+10)	0	0/(0+4)	0	0/(0+1)	0	0/(0+4)	0	0/(0+1)	0	0/(0+1)	0
Norfloxacin	0/(0+7)	0	1/(1+4)	20	0/(0+2)	0	0/(0+10)	0	0/(0+4)	0	0/(0+1)	0	0/(0+4)	0	0/(0+1)	0	0/(0+1)	0
Rifampicin	0/(0+7)	0	1/(1+4)	20	0/(0+2)	0	0/(0+10)	0	0/(0+4)	0	0/(0+2)	0	0/(0+4)	0	0/(0+1)	0	0/(0+1)	0

*MRSA, Methicillin resistant staphylococcus aureus; P. aeruginosa, Pseudomonas aeruginosa; E-coli, Escherichia coli; S-milleri, Streptococcus milleri*.

**Figure 3:**
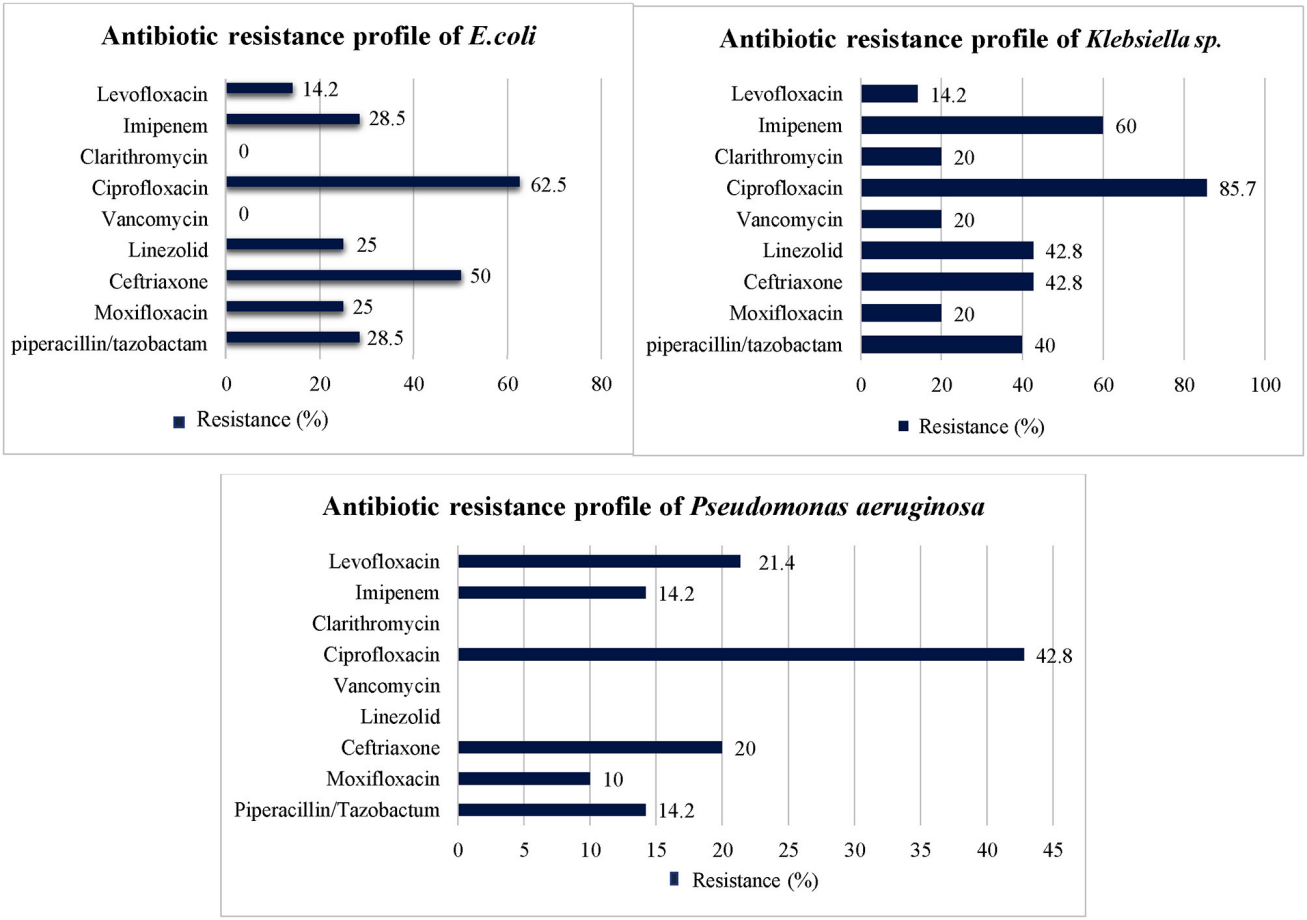
Antibiotic resistance profile of three most prevalent bacterial species.

The antibiotics resistance profile of the three most prevalent bacterial species against the commonly used antibiotics at the start of the treatment is presented in Figure 3.

### Modification in Antibiotic Therapy Among the Patients

Among the total 110 patients, antibiotic therapy was modified in 52 (47.3%) patients based on either clinician's judgement and/or culture and sensitivity results. Out of 52 patients, initial treatment was replaced with imipenem in 20 (38.4%) patients, piperacillin/tazobactam in 13 (25%) patients and linezolid in 12 (23%) patients (Table 4).

**Table 4:** Modification in antibiotic therapy based on clinician's suggestion and culture and sensitivity results (*N* = 110).

**Modified therapy**	**Modified after positive culture *n* (%)**
Piperacillin+ tazobactam	13 (25)
Moxifloxacin	2 (3.8)
Ceftriaxone	5 (9.6)
Linezolid	12 (23)
Amikacin	9 (17.3)
Ciprofloxacin	10 (19.2)
Vancomycin	8 (15.3)
Imipenem	20 (38.4)
Doxycycline	2 (3.8)

### Treatment Outcomes Among the Patients

In the total 110 patients, 82 (74.5%) completed their treatment with clinical improvement, 12 (10.9%) showed no clinical improvement at any stage of treatment, five (4.5%) were lost to follow up and 11 (10%) died before treatment completion (Table 5).

**Table 5:** Treatment outcomes among the empyema thoracis patients (*N* = 110).

**Treatment outcomes**	**Patients, *n* (%)**	***n* (%)**
**Successful**		**82 (74.5)**
Treatment completed with clinical improvement	82 (74.5)	
**Unsuccessful**		**28 (25.5)**
No clinical improvement	12 (11)	
Loss to follow up	5 (4.5)	
Death	11 (10)	

When treatment outcomes with respect to patient's characteristics, a statistically significant difference was found in male (i.e., *p* < 0.05) with respect to loss to follow up. Likewise, statistically significant difference was found in biomass (i.e., *p* < 0.05) with regard to treatment completed with clinical improvement. Similarly, statistically significant difference was found in improved with empiric therapy (i.e., *p* < 0.05) with regard to treatment completed with clinical improvement and death (Table 6).

**Table 6:** Treatment outcomes with regard to patient characteristics.

**Characteristics**	**Outcome**								
	**Treatment completed with clinical improvement** ***n =* 82**	***p*-value**	**No clinical improvement** ***n =* 12**	***p*-value**	**Loss to follow up *n =* 5**	***p*-value**	**Death *n =* 11**	***p*-value**	**Total *n =* 110**
**Gender**		0.150		0.441		**0.047**		0.733	
Male	59 (78.6)		7 (9.3)		1 (1.3)		8 (10.6)		75 (100)
Female	23 (65.7)		5 (14.2)		4 (11.4)		3 (8.5)		35 (100)
**Residence**		0.591		0.686		0.449		0.512	
Urban	31 (77.5)		5 (12.5)		1 (2.5)		3 (7.5)		40 (100)
Rural	51 (72.8)		7 (10)		4 (5.7)		8 (11.4)		70 (100)
**Smoking**		0.344		0.506		0.810		0.607	
Current smoker	7 (77.7)		0 (0)		1 (11.1)		1 (11.1)		9 (100)
Ex- smoker	15 (83.3)		2 (11.1)		0 (0)		1 (5.5)		18 (100)
Non- smoker	60 (72.2)		10 (12.0)		4 (4.8)		9 (10.8)		83 (100)
**Biomass fuel exposure**		**0.023**		0.271		0.067		0.468	
Yes	24 (61.5)		6 (15.3)		4 (10.2)		5 (12.8)		39 (100)
No	58 (81.6)		6 (8.4)		1 (1.4)		6 (8.4)		71 (100)
**Diagnosis**		0.289		0.464		0.453		0.218	
Hospital acquired empyema	18 (78.2)		2 (8.6)		2 (8.6)		1 (4.3)		23 (100)
Community acquired empyema	59 (71.9)		10 (12.1)		3 (3.6)		10 (12.1)		82 (100)
Tuberculosis empyema	5 (100)		0 (0)		0 (0)		0 (0)		5 (100)
**Microorganism growth**		0.440		0.841		0.242		0.088	
Positive	45 (77.5)		6 (10.3)		4 (6.8)		3 (5.1)		58 (100)
Negative	36 (75)		6 (12.5)		1 (2.0)		5 (10.4)		48 (100)
Not tested	1 (24)		0 (0)		0 (0)		3 (75)		4 (100)
**Microorganism involved**		0.449		0.780		0.510		0.213	
Monomicrobial	39 (78)		5 (10)		3 (6)		3 (6)		50 (100)
Polymicrobial	6 (75)		1 (12.5)		1 (12.5)		0 (0)		8 (100)
No growth seen	36 (75)		6 (12.5)		1 (2.0)		5 (10.4)		48 (100)
Not tested	1 (25)		0 (0)		0 (0)		3 (75)		4 (100)
**Improvement in symptoms**		**<** **0.0005**		0.999		0.900		**0.043**	
Improved after modification	38 (95)		0 (0)		2 (5)		0 (0)		40 (100)
Improved with empiric therapy	44 (93.6)		0 (0)		2 (4.2)		1 (2.1)		47 (100)
Not improved	0 (0)		12 (52.1)		1 (4.3)		10 (43.4)		23 (100)
**Duration of symptoms**		0.667		0.730		0.536		0.486	
<2 weeks	20 (71.4)		3 (10.7)		2 (7.1)		3 (10.7)		28 (100)
2–4 weeks	39 (76.4)		5 (9.8)		3 (5.8)		4 (7.8)		51 (100)
More than 4 weeks	23 (74.1)		4 (12.9)		0 (0)		4 (12.9)		31 (100)
**Resistance to 5 antibiotic classes**		0.889		0.793		0.319		0.437	
Yes	31 (73.8)		5 (11.9)		3 (7.1)		3 (7.1)		42 (100)
No	51 (75)		7 (10.2)		2 (2.9)		8 (11.7)		68 (100)
**Therapy modified**		0.917		0.841		0.170		0.174	
Yes	39 (75)		6 (11.5)		4 (7.6)		3 (5.7)		52 (100)
No	43 (74.1)		6 (10.3)		1 (1.7)		8 (13.7)		58 (100)

*p-value less than 0.05 in bold*.

Treatment modification with imipenem and amikacin + imipenem lead to death in more case (a total of 8 and 4 died, respectively) as compared to other modification. Number of deaths in culture negative patients (5 out of 48) was slightly higher than culture positive (3 out of 58) patients. Number of deaths with regard to modified treatment and number of deaths with regard to culture status is provided in Supplementary Tables 3, 4, respectively.

### Factors Associated With Unsuccessful Treatment Outcomes Among the Patients

In multivariate binary logistic regression analysis, none of the patient's variables were significantly associated with unsuccessful treatment outcomes (Table 7).

**Table 7:** Factors associated with unsuccessful treatment outcome: multivariate binary logistic regression analysis.

	**Univariate analysis**		**Multivariate analysis**	
**Variable**	***p*-value**	**OR (95% CI)**	***p*-value**	**AOR (95%CI)**
Biomass fuel exposure	**0.023**	2.788 (1.154 to 6.736)	0.071	2.333 (0.931 to 5.842)
Empiric therapy with piperacillin/tazobactam	**0.037**	0.390 (0.161 to 0.943)	0.113	0.473 (0.187 to 1.195)
Empiric therapy with moxifloxacin	**0.041**	2.568 (1.039 to 6.344)	0.170	1.946 (0.751 to 5.041)

*Hosmer and Lemeshow test (4.534), p = 0.475; Nagelkerke R Square (0.136); Model summary = Chi square (10.638), df (3), p = 0.014. p-value less than 0.05 in bold*.

### Factors Associated With Duration of Hospital Stay Among the Patients

In multivariate linear regression, the factors which were associated significantly with duration of hospital stay included; duration of symptoms <2 weeks prior to admission (beta = −0.247, *p* = 0.008) and resistance to five antibiotic classes (beta = 0.280, *p* = 0.02) (Table 8).

**Table 8:** Factors associated with duration of hospital stay: linear regression analysis.

	**Univariate analysis**			Multivariate analysis^*^		
**Variable**	***p*-value**	**S. E**	**B**	***p*-value**	**S. E**	**B**
Monomicrobial growth	**0.006**	0.258	0.259	0.639	0.404	0.069
Gram- negative organism	**0.019**	0.261	−0.224	0.764	0.453	−0.047
Duration of symptoms <2 weeks	**0.001**	0.290	−0.312	**0.008**	0.291	−0.247
Resistance to 5 antibiotic classes	** <0.0005**	0.339	0.257	**0.02**	0.344	0.280

* *Nagelkerke R Square (0.132); Model summary = Chi square (10.295), df (4), p = 0.036. p-value less than 0.05 in bold*.

## Discussion

Empyema thoracis is a rare complication of the pleural space of lungs (31), and there is a scarcity of data, especially from developing countries, due to unavailability of published studies (6, 8). To the extent of our knowledge, this is the first study from Pakistan that concurrently evaluated the spectrum of microorganism, antibiotic resistance pattern, duration of hospital stay, and treatment outcomes among the patients with empyema thoracis. Evaluation of antibiotic resistance pattern, spectrum of microorganisms and treatment outcomes in the empyema thoracis patients could be helpful for the clinicians in prescribing appropriate antibiotics for achieving better clinical outcomes. In this study cohort, close to 25% of the patients did not complete the treatment successfully. Most common gram- negative bacterial isolates showed high level of resistance against empiric therapy. We also analyzed the independent factors associated with unsuccessful treatment outcome and length of hospital stay.

In the present study, most of the patients with empyema thoracis (68.2%) were male. This finding is consistent with other similar studies conducted in Pakistan (66.1%), India (75.4%) and Canada (64%) (25, 32, 33). The probable reason due to which the males were more vulnerable to disease is having only one copy of X-linked genes responsible for production of immunoglobulins (24, 34). In this study, 58 (52.7%) patients were culture positive. Contrary to this, a study conducted in the US (43%) reported relatively low culture positivity rate (35). Whereas, studies conducted in India (87.2%) and China (68%) showed high percentages (36, 37). The possible reason for low culture positivity might be use of antibiotics prior to sample collection for culture sensitivity test, improper specimen transportation, wrong techniques of sample collection and/or other viral, parasitic and fungal infections (24, 38).

In this study cohort, *Pseudomonas* (18.1%), and *Klebsiella sp*. (10%) were the most commonly identified gram- negative isolates. Similarly, the same results were reported by a study conducted in India (8). For the treatment of empyema thoracis, the physicians in our study prescribed piperacillin/tazobactam alone or in combination with cephalosporins, carbapenems and fluoroquinolones which in line with AATS guidelines. But the most common bacterial isolates identified in this study showed high level of resistance against aforementioned antibiotics. Similarly, other studies conducted in Pakistan, India, Hungary, and the US also reported high level of resistance to these antibiotics (39–43). The probable reason for increased antibiotic resistance could be an excess, inappropriate use and careless selection of antibiotics (44), as a number of studies have reported irrational prescribing, dispensing and use of antibiotics in Pakistan (45–48). The rapidly growing antibiotic resistance in Pakistan demand effective implementation of antibiotic stewardship program in accordance with the recommendations of the WHO (49–51). In addition, promotion of One Health, which involves collaboration of all the healthcare professionals responsible for antibiotic use in humans, animals and environment, seems mandatory to halt gradual extinction of effective antibiotics.

It is an important point to notice that in the present study, despite of being aware of the most commonly identified bacterial isolates and their resistance pattern, the physicians neither tailored the antibiotic treatment according to the need of the patient nor according to the culture and sensitivity results. The AATS guidelines recommends piperacillin/tazobactam, cephalosporins, carbapenems and fluoroquinolones to be taken as empiric therapy for empyema thoracis (2), however, this may not be a viable choice here because the most common bacterial isolates showed high level of resistance against these antibiotics (Tables 3, 4). However, amoxicillin/clavulanic acid, ertapenem, clarithromycin, colistin, vancomycin, fosfomycin, rifampicin and tigecycline showed better sensitivity against most common bacterial isolates. Studies from other countries like the US, Italy and Germany also supported the judicious use of aforementioned antibiotics (35, 52–54). The German guideline by The Paul- Ehrlich- Gesellschaft fur Chemotherapie have also suggested the use of amoxicillin/clavulanic acid, ertapenem, clarithromycin, colistin, vancomycin, fosfomycin, rifampicin and tigecycline against most common gram- negative bacterial isolates causing empyema thoracis (54, 55). On the basis of the antibacterial spectrum of these antibiotics against most commonly identified bacterial isolates, vancomycin in combination with amoxicillin/clavulanic acid, sulbactam or clarithromycin and colistin alone or in combination with tigecycline, fosfomycin, rifampicin, sulbactam, or ertapenem seems to be the better option for most common bacterial isolates (52, 54).

With regard to final treatment outcomes, the treatment success rate in this study was 74.5%. Similarly, high success rate was reported in a study from the United Kingdom (UK) (88%) (56). The probable reason for high success rate in the UK study might be due to the less severity of the disease and use of targeted antibiotics earlier (56, 57). We found that the mortality rate in this study was 10% which was comparable with the study of Netherland (8%) (58). However, another study conducted in Pakistan reported relatively less mortality rate (1.3%) (25). Contrary to this, China reported higher mortality rate (33.3%) (59). The major reason for high mortality rate could be poor diagnosis, delay in empiric therapy, resistance to antibiotics, longer duration of hospital stay, harmful habits like smoking, and comorbidities (59). Moreover, a study conducted in the UK showed increased hospital stay (>2 weeks) (56). While, the mean length of hospital stay in our study was 2.3 weeks and these results were almost similar to a study conducted in India (2 weeks) (60). This Indian study showed that shorter duration of hospital stay was observed among those patients who responded better toward their treatment (60).

Our study found that resistance to multiple antibiotics increased the duration of hospital stay and these results were comparable with a study conducted in Vietnam (61). The possible reason for prolonged hospital stay due to multidrug resistance may be due to the fact that such patients have lesser treatment options and clinicians have to repeatedly switch the antibiotic therapy in search of appropriate antibiotics. This study further showed that patients with duration of symptoms <2 weeks prior to hospital visit had less stay in hospital. The probable reasons for lesser stay in hospital in such patients could be the early diagnosis of the disease, and the start of antibiotics (57, 60).

This study has some limitations. First, this study was a single centered study and did not describe the data of the whole country. However, it describes the antibiotic use and resistance pattern, and treatment outcomes in a tertiary care hospital which serves a large population living in the Southern part of the Punjab province of Pakistan. We assume that antibiotic resistance and sensitivity pattern may be similar for whole of the Punjab province of Pakistan. However, multicenter studies with larger sample size are required to warrant this statement. Second, because empyema thoracis is a rare disease, we were unable to gather data from a larger sample. Third, though a valid and widely used statistical analysis was performed to answer the study objectives, a hierarchical cluster analysis was not performed due to unavailability of data in required format which is recommended in future studies.

## Conclusion

Close to one fourth of the patients did not complete their treatment successfully. *Pseudomonas aeruginosa, Klebsiella sp., and E. coli* were the most common bacterial isolates identified in the study cohort. Most of the bacterial isolates showed resistance against empiric treatment. Given the antibacterial spectrum of the tested antibiotics, the combination of vancomycin with amoxicillin/clavulanic acid, sulbactam or clarithromycin, and colistin alone or in combination with other antibiotics like tigecycline, sulbactam, rifampicin, fosfomycin or ertapenem may be considered as the treatment options.

## Data Availability Statement

The original contributions presented in the study are included in the article/Supplementary Material, further inquiries can be directed to the corresponding author/s.

## Author Contributions

MA, MN, SS, and SM: conceptualization, formal analysis, methodology, and validation. MN: data curation. MA: supervision. MA, MN, SS, SM, MH, IM, MNI, and NA: writing—original draft and writing—review and editing. All authors contributed to the article and approved the submitted version.

## Conflict of Interest

The authors declare that the research was conducted in the absence of any commercial or financial relationships that could be construed as a potential conflict of interest.

## Publisher's Note

All claims expressed in this article are solely those of the authors and do not necessarily represent those of their affiliated organizations, or those of the publisher, the editors and the reviewers. Any product that may be evaluated in this article, or claim that may be made by its manufacturer, is not guaranteed or endorsed by the publisher.
