# Supplementary material for: Spectrum of Microorganisms, Antibiotic Resistance Pattern, and Treatment Outcomes Among Patients With Empyema Thoracis: A Descriptive Cross-Sectional Study From the Bahawal Victoria Hospital Bahawalpur, Punjab, Pakistan
Source: Front Med (Lausanne). 2021 Aug 6;8:665963. doi: 10.3389/fmed.2021.665963 (PMC8377472; doi:10.3389/fmed.2021.665963)
Supplement: Supplementary file 1 [file Table_1.DOCX]

**Supplementary File 1: Number of resistant drugs with regard to identified organisms**

| **Organism isolates** | **Number of antibiotics** | |
| --- | --- | --- |
| **Gram-negative** | **Minimum** | **Maximum** |
| *Escherichia coli* | 1 | 26 |
| *Klebsiella* | 1 | 48 |
| *Acinetobacter* | 1 | 18 |
| *Pseudomonas aeruginosa* | 1 | 20 |
| *Enterobacter* | 1 | 9 |
| *Proteus mirabilis* | 1 | 3 |
| **Gram- positive** |  |  |
| *Staphylococcus aureus* | 1 | 2 |
| *MRSA* | 1 | 6 |
| *S. milleri* | 2 | - |
| ***Mycobacterium tuberculosis*** | 0 | 0 |
