# Supplementary material for: Spectrum of Microorganisms, Antibiotic Resistance Pattern, and Treatment Outcomes Among Patients With Empyema Thoracis: A Descriptive Cross-Sectional Study From the Bahawal Victoria Hospital Bahawalpur, Punjab, Pakistan
Source: Front Med (Lausanne). 2021 Aug 6;8:665963. doi: 10.3389/fmed.2021.665963 (PMC8377472; doi:10.3389/fmed.2021.665963)
Supplement: Supplementary file 2 [file Table_2.DOCX]

**Supplementary File 2: Drug resistance index for E. coli, Klebsiella and Pseudomonas**

**Drug resistance index (DRI) for *E-coli***

**Step 1: Resistance data**

***1^st^ 6- months***

| **Time**  **Period** | **Species** | **Drug Class** | **Isolates, TOTAL** | **Isolates,**  **RES** | **RES**  **RATE** |
| --- | --- | --- | --- | --- | --- |
| 2019 | *E.coli* | Penicillins | 40 | 6 | 0.15 |
| 2019 | *E.coli* | Carbapenems | 19 | 3 | 0.15 |
| 2019 | *E.coli* | Cephalosporins | 56 | 5 | 0.08 |
| 2020 | *E.coli* | Quinolones | 42 | 5 | 0.11 |
| 2020 | *E.coli* | Aminoglycosides | 18 | 1 | 0.05 |
| 2020 | *E.coli* | Others | 120 | 6 | 0.05 |

***2^nd^ 6- months***

| **Time**  **Period** | **Species** | **Drug Class** | **Isolates, TOTAL** | **Isolates,**  **RES** | **RES**  **RATE** |
| --- | --- | --- | --- | --- | --- |
| 2019 | *E.coli* | Penicillins | 20 | 8 | 0.4 |
| 2019 | *E.coli* | Carbapenems | 10 | 2 | 0.2 |
| 2019 | *E.coli* | Cephalosporins | 29 | 16 | 0.55 |
| 2020 | *E.coli* | Quinolones | 17 | 7 | 0.41 |
| 2020 | *E.coli* | Aminoglycosides | 9 | 3 | 0.33 |
| 2020 | *E.coli* | Others | 46 | 6 | 0.13 |

**Step 2: Utilization data**

***1^st^ 6- months***

| **Time**  **Period** | **Drug Class** | **Use** | **TOTAL PERIOD USE** | **USE**  **WEIGHT** |
| --- | --- | --- | --- | --- |
| 2019 | Penicillins | 5 | 34 | 0.14 |
| 2019 | Carbapenems | 4 | 34 | 0.11 |
| 2019 | Cephalosporins | 7 | 34 | 0.20 |
| 2020 | Quinolones | 10 | 34 | 0.29 |
| 2020 | Aminoglycosides | 4 | 34 | 0.11 |
| 2020 | Others | 4 | 34 | 0.11 |

***2^nd^ 6- months***

| **Time**  **Period** | **Drug Class** | **Use** | **TOTAL PERIOD USE** | **USE**  **WEIGHT** |
| --- | --- | --- | --- | --- |
| 2019 | Penicillins | 1 | 3 | 0.3 |
| 2019 | Carbapenems | 0 | 3 | 0 |
| 2019 | Cephalosporins | 0 | 3 | 0 |
| 2020 | Quinolones | 1 | 3 | 0.3 |
| 2020 | Aminoglycosides | 0 | 3 | 0 |
| 2020 | Others | 1 | 3 | 0.3 |

**Step 3: Index calculations**

***1^st^ 6- months***

| **Time**  **Period** | **Species** | **Drug Class** | **RES**  **RATE** | **USE**  **WEIGHT** | **WEIGHTED RESISTANCE** | **INDEX**  **VALUE** |
| --- | --- | --- | --- | --- | --- | --- |
| 2019 | *E.coli* | Penicillins | 0.15 | 0.14 | 0.021 | 0.094 |
| 2019 | *E.coli* | Carbapenems | 0.15 | 0.11 | 0.016 | 0.094 |
| 2019 | *E.coli* | Cephalosporins | 0.08 | 0.20 | 0.016 | 0.094 |
| 2020 | *E.coli* | Quinolones | 0.11 | 0.29 | 0.031 | 0.094 |
| 2020 | *E.coli* | Aminoglycosides | 0.05 | 0.11 | 0.005 | 0.094 |
| 2020 | *E.coli* | Others | 0.05 | 0.11 | 0.005 | 0.094 |

***2^nd^ 6- months***

| **Time**  **Period** | **Species** | **Drug Class** | **RES**  **RATE** | **USE**  **WEIGHT** | **WEIGHTED RESISTANCE** | **INDEX**  **VALUE** |
| --- | --- | --- | --- | --- | --- | --- |
| 2019 | *E.coli* | Penicillins | 0.4 | 0.3 | 0.7 | 0.86 |
| 2019 | *E.coli* | Carbapenems | 0.2 | 0 | 0 | 0.86 |
| 2019 | *E.coli* | Cephalosporins | 0.55 | 0 | 0 | 0.86 |
| 2020 | *E.coli* | Quinolones | 0.41 | 0.3 | 0.123 | 0.86 |
| 2020 | *E.coli* | Aminoglycosides | 0.33 | 0 | 0 | 0.86 |
| 2020 | *E.coli* | Others | 0.13 | 0.3 | 0.039 | 0.86 |

**Drug resistance index (DRI) for *Klebsiella***

**Step 1: Resistance data**

***1^st^ 6- months***

| **Time**  **Period** | **Species** | **Drug Class** | **Isolates, TOTAL** | **Isolates,**  **RES** | **RES**  **RATE** |
| --- | --- | --- | --- | --- | --- |
| 2019 | *Klebsiella* | Penicillins | 42 | 7 | 0.16 |
| 2019 | *Klebsiella* | Carbapenems | 18 | 7 | 0.38 |
| 2019 | *Klebsiella* | Cephalosporins | 53 | 8 | 0.15 |
| 2020 | *Klebsiella* | Quinolones | 41 | 7 | 0.17 |
| 2020 | *Klebsiella* | Aminoglycosides | 18 | 5 | 0.27 |
| 2020 | *Klebsiella* | Others | 115 | 3 | 0.02 |

***2^nd^ 6- months***

| **Time**  **Period** | **Species** | **Drug Class** | **Isolates, TOTAL** | **Isolates,**  **RES** | **RES**  **RATE** |
| --- | --- | --- | --- | --- | --- |
| 2019 | *Klebsiella* | Penicillins | 15 | 15 | 1 |
| 2019 | *Klebsiella* | Carbapenems | 10 | 7 | 0.7 |
| 2019 | *Klebsiella* | Cephalosporins | 29 | 18 | 0.62 |
| 2020 | *Klebsiella* | Quinolones | 19 | 13 | 0.68 |
| 2020 | *Klebsiella* | Aminoglycosides | 11 | 10 | 0.90 |
| 2020 | *Klebsiella* | Others | 50 | 2 | 0.56 |

**Step 2: Utilization data**

***1^st^ 6- months***

| **Time**  **Period** | **Drug Class** | **Use** | **TOTAL PERIOD USE** | **USE**  **WEIGHT** |
| --- | --- | --- | --- | --- |
| 2019 | Penicillins | 10 | 34 | 0.29 |
| 2019 | Carbapenems | 5 | 34 | 0.14 |
| 2019 | Cephalosporins | 5 | 34 | 0.14 |
| 2020 | Quinolones | 5 | 34 | 0.14 |
| 2020 | Aminoglycosides | 3 | 34 | 0.08 |
| 2020 | Others | 6 | 34 | 0.17 |

***2^nd^ 6- months***

| **Time**  **Period** | **Drug Class** | **Use** | **TOTAL PERIOD USE** | **USE**  **WEIGHT** |
| --- | --- | --- | --- | --- |
| 2019 | Penicillins | 1 | 3 | 0.3 |
| 2019 | Carbapenems | 1 | 3 | 0.3 |
| 2019 | Cephalosporins | 0 | 3 | 0 |
| 2020 | Quinolones | 1 | 3 | 0.3 |
| 2020 | Aminoglycosides | 0 | 3 | 0 |
| 2020 | Others | 0 | 3 | 0 |

**Step 3: Index calculations**

***1^st^ 6- months***

| **Time**  **Period** | **Species** | **Drug Class** | **RES**  **RATE** | **USE**  **WEIGHT** | **WEIGHTED RESISTANCE** | **INDEX**  **VALUE** |
| --- | --- | --- | --- | --- | --- | --- |
| 2019 | *Klebsiella* | Penicillins | 0.16 | 0.29 | 0.046 | 0.16 |
| 2019 | *Klebsiella* | Carbapenems | 0.38 | 0.14 | 0.053 | 0.16 |
| 2019 | *Klebsiella* | Cephalosporins | 0.15 | 0.14 | 0.021 | 0.16 |
| 2020 | *Klebsiella* | Quinolones | 0.17 | 0.14 | 0.023 | 0.16 |
| 2020 | *Klebsiella* | Aminoglycosides | 0.27 | 0.08 | 0.021 | 0.16 |
| 2020 | *Klebsiella* | Others | 0.02 | 0.17 | 0.0034 | 0.16 |

***2^nd^ 6- months***

| **Time**  **Period** | **Species** | **Drug Class** | **RES**  **RATE** | **USE**  **WEIGHT** | **WEIGHTED RESISTANCE** | **INDEX**  **VALUE** |
| --- | --- | --- | --- | --- | --- | --- |
| 2019 | *Klebsiella* | Penicillins | 1 | 0.3 | 0.3 | 0.7 |
| 2019 | *Klebsiella* | Carbapenems | 0.7 | 0.3 | 0.21 | 0.7 |
| 2019 | *Klebsiella* | Cephalosporins | 0.62 | 0 | 0 | 0.7 |
| 2020 | *Klebsiella* | Quinolones | 0.68 | 0.3 | 0.20 | 0.7 |
| 2020 | *Klebsiella* | Aminoglycosides | 0.90 | 0 | 0 | 0.7 |
| 2020 | *Klebsiella* | Others | 0.56 | 0 | 0 | 0.7 |

**Drug resistance index (DRI) for *Pseudomonas aeruginosa***

**Step 1: Resistance data**

***1^st^ 6- months***

| **Time**  **Period** | **Species** | **Drug Class** | **Isolates, TOTAL** | **Isolates,**  **RES** | **RES**  **RATE** |
| --- | --- | --- | --- | --- | --- |
| 2019 | *Pseudomonas* | Penicillins | 42 | 2 | 0.04 |
| 2019 | *Pseudomonas* | Carbapenems | 18 | 1 | 0.05 |
| 2019 | *Pseudomonas* | Cephalosporins | 60 | 7 | 0.11 |
| 2020 | *Pseudomonas* | Quinolones | 38 | 3 | 0.07 |
| 2020 | *Pseudomonas* | Aminoglycosides | 18 | 2 | 0.11 |
| 2020 | *Pseudomonas* | Others | 119 | 1 | 0.008 |

***2^nd^ 6- months***

| **Time**  **Period** | **Species** | **Drug Class** | **Isolates, TOTAL** | **Isolates,**  **RES** | **RES**  **RATE** |
| --- | --- | --- | --- | --- | --- |
| 2019 | *Pseudomonas* | Penicillins | 65 | 5 | 0.07 |
| 2019 | *Pseudomonas* | Carbapenems | 37 | 10 | 0.27 |
| 2019 | *Pseudomonas* | Cephalosporins | 92 | 28 | 0.30 |
| 2020 | *Pseudomonas* | Quinolones | 68 | 15 | 0.22 |
| 2020 | *Pseudomonas* | Aminoglycosides | 40 | 24 | 0.6 |
| 2020 | *Pseudomonas* | Others | 167 | 5 | 0.02 |

**Step 2: Utilization data**

***1^st^ 6- months***

| **Time**  **Period** | **Drug Class** | **Use** | **TOTAL PERIOD USE** | **USE**  **WEIGHT** |
| --- | --- | --- | --- | --- |
| 2019 | Penicillins | 16 | 58 | 0.27 |
| 2019 | Carbapenems | 12 | 58 | 0.20 |
| 2019 | Cephalosporins | 6 | 58 | 0.10 |
| 2020 | Quinolones | 14 | 58 | 0.24 |
| 2020 | Aminoglycosides | 3 | 58 | 0.05 |
| 2020 | Others | 7 | 58 | 0.12 |

***2^nd^ 6- months***

| **Time**  **Period** | **Drug Class** | **Use** | **TOTAL PERIOD USE** | **USE**  **WEIGHT** |
| --- | --- | --- | --- | --- |
| 2019 | Penicillins | 1 | 7 | 0.14 |
| 2019 | Carbapenems | 1 | 7 | 0.14 |
| 2019 | Cephalosporins | 1 | 7 | 0.14 |
| 2020 | Quinolones | 2 | 7 | 0.28 |
| 2020 | Aminoglycosides | 1 | 7 | 0.14 |
| 2020 | Others | 1 | 7 | 0.14 |

**Step 3: Index calculations**

***1^st^ 6- months***

| **Time**  **Period** | **Species** | **Drug Class** | **RES**  **RATE** | **USE**  **WEIGHT** | **WEIGHTED RESISTANCE** | **INDEX**  **VALUE** |
| --- | --- | --- | --- | --- | --- | --- |
| 2019 | *Pseudomonas* | Penicillins | 0.04 | 0.27 | 0.010 | 0.065 |
| 2019 | *Pseudomonas* | Carbapenems | 0.05 | 0.20 | 0.01 | 0.065 |
| 2019 | *Pseudomonas* | Cephalosporins | 0.11 | 0.10 | 0.024 | 0.065 |
| 2020 | *Pseudomonas* | Quinolones | 0.07 | 0.24 | 0.016 | 0.065 |
| 2020 | *Pseudomonas* | Aminoglycosides | 0.11 | 0.05 | 0.005 | 0.065 |
| 2020 | *Pseudomonas* | Others | 0.008 | 0.12 | 0.00096 | 0.065 |

***2^nd^ 6- months***

| **Time**  **Period** | **Species** | **Drug Class** | **RES**  **RATE** | **USE**  **WEIGHT** | **WEIGHTED RESISTANCE** | **INDEX**  **VALUE** |
| --- | --- | --- | --- | --- | --- | --- |
| 2019 | *Pseudomonas* | Penicillins | 0.07 | 0.14 | 0.0098 | 0.23 |
| 2019 | *Pseudomonas* | Carbapenems | 0.27 | 0.14 | 0.037 | 0.23 |
| 2019 | *Pseudomonas* | Cephalosporins | 0.30 | 0.14 | 0.042 | 0.23 |
| 2020 | *Pseudomonas* | Quinolones | 0.22 | 0.28 | 0.061 | 0.23 |
| 2020 | *Pseudomonas* | Aminoglycosides | 0.6 | 0.14 | 0.084 | 0.23 |
| 2020 | *Pseudomonas* | Others | 0.02 | 0.14 | 0.0028 | 0.23 |
