# Supplementary material for: Spectrum of Microorganisms, Antibiotic Resistance Pattern, and Treatment Outcomes Among Patients With Empyema Thoracis: A Descriptive Cross-Sectional Study From the Bahawal Victoria Hospital Bahawalpur, Punjab, Pakistan
Source: Front Med (Lausanne). 2021 Aug 6;8:665963. doi: 10.3389/fmed.2021.665963 (PMC8377472; doi:10.3389/fmed.2021.665963)
Supplement: Supplementary file 3 [file Table_3.DOCX]

**Supplementary File 3: Number of deaths with regard to modified treatment**

| **Modified treatment** | **Death** | | **Total** |
| --- | --- | --- | --- |
|  | **No** | **Yes** |  |
| Piperacillin/ tazobactam | 3 | 0 | 3 |
| Piperacillin/ tazobactam + linezolid | 3 | 0 | 3 |
| Piperacillin/ tazobactam +ciprofloxacin | 5 | 0 | 5 |
| Piperacillin/ tazobactam + vancomycin | 1 | 0 | 1 |
| Piperacillin/ tazobactam + linezolid + vancomycin | 1 | 0 | 1 |
| Moxifloxacin | 1 | 0 | 1 |
| Moxifloxacin + ceftriaxone | 1 | 0 | 1 |
| Ceftriaxone | 2 | **1** | 3 |
| Ceftriaxone + ciprofloxacin | 1 | 0 | 1 |
| Linezolid | 1 | 0 | 1 |
| Linezolid + vancomycin | 2 | 0 | 2 |
| Linezolid + ciprofloxacin | 1 | 0 | 1 |
| Linezolid + imipenem | 2 | **1** | 3 |
| Linezolid + piperacillin/ tazobactam + vancomycin | 1 | 0 | 1 |
| Linezolid + amikacin + imipenem | 1 | 0 | 1 |
| Amikacin | 3 | 0 | 3 |
| Amikacin + vancomycin | 1 | 0 | 1 |
| Amikacin + imipenem | 4 | 0 | 4 |
| Ciprofloxacin | 1 | 0 | 1 |
| Ciprofloxacin + imipenem | 2 | 0 | 2 |
| Vancomycin | 3 | 0 | 3 |
| Imipenem | 7 | **1** | 8 |
| Imipenem + doxycycline | 1 | 0 | 1 |
| Doxycycline | 1 | 0 | 1 |
| **Total** | **49** | **3** | **52** |
