# Supplementary material for: Spectrum of Microorganisms, Antibiotic Resistance Pattern, and Treatment Outcomes Among Patients With Empyema Thoracis: A Descriptive Cross-Sectional Study From the Bahawal Victoria Hospital Bahawalpur, Punjab, Pakistan
Source: Front Med (Lausanne). 2021 Aug 6;8:665963. doi: 10.3389/fmed.2021.665963 (PMC8377472; doi:10.3389/fmed.2021.665963)
Supplement: Supplementary file 4 [file Table_4.DOCX]

**Supplementary File 4: Number of deaths with regard to culture status**

| **Culture status** | **Death** | | **Total** |
| --- | --- | --- | --- |
|  | **No** | **Yes** |  |
| Positive | 55 | 3 | 58 |
| Negative | 43 | 5 | 48 |
| Specimen not sent for culture | 1 | 3 | 4 |
| **Total** | **99** | **11** | **110** |
